# A Case Report of Right Atrial Thrombosis Complicated by Multiple Pulmonary Emboli: POCUS For the Win!

**DOI:** 10.21980/J8TM07

**Published:** 2025-01-31

**Authors:** Andrea Wolff, Evan Leibner, Jill Gualdoni

**Affiliations:** * Creighton University School of Medicine, Dignity Health East Valley, Department of Emergency Medicine, Chandler, AZ; ^ Creighton University School of Medicine, Dignity Health East Valley, Department of Internal Medicine, Chandler, AZ

## Abstract

A 78-year-old gentleman presented to the emergency department (ED) for palpitations and dizziness. He had a complicated medical history including atrial fibrillation (AF), recently status post a Watchman procedure, oxygen-dependent chronic obstructive pulmonary disease (COPD), and heart failure with preserved ejection fraction (HFpEF). Point-of-care ultrasound (POCUS) revealed the presence of an intracardiac right atrial thrombus. Computed tomography (CT) angiography confirmed the presence of multiple pulmonary emboli (PE), and extension of the thrombus into the inferior vena cava. Pulmonary emboli are a common complication of thrombus in the right atrium. Management may include anticoagulation, thrombolysis, or thrombectomy. This case highlights that emergency physicians can expedite the diagnosis of intracardiac thrombus by using POCUS. The case presented describes a medically complex patient presenting with symptomatic right intracardiac and inferior vena caval thrombosis complicated by multiple PE. Point-of care ultrasound of the heart and lungs were included in his initial assessment, revealing findings of an intracardiac thrombus, and ruling out multiple other differential diagnoses including pericardial tamponade, pleural effusion, pulmonary edema, and pneumothorax. This finding changed the trajectory of this patient’s evaluation and management, and demonstrates the important role of POCUS in the care of ED patients with undifferentiated cardiopulmonary symptoms.

**Topics:**

Point-of care ultrasound (POCUS), focused cardiac ultrasound (FOCUS), inferior vena cava thrombosis, right atrial thrombosis, pulmonary embolism, computed tomography, echocardiography.

## Brief introduction

Point of care ultrasound (POCUS) is a valuable diagnostic and procedural tool in the hands of emergency medicine (EM) clinicians, and focused cardiac ultrasound (FOCUS) continues to gain traction in the evaluation of patients with suspected cardiopulmonary disease. The diagnoses of pericardial effusion, pericardial tamponade, and cardiogenic shock are commonly made by EM physicians with bedside ultrasonography.^1^,^2^,^3^ However, diagnosis of pulmonary emboli (PE) is more challenging.

Considering that PE is the third most common cause of cardiovascular death in the United States, with an incidence of 117 cases per 100,000 people annually,^4^,^5^ familiarity with the sonographic findings associated with this diagnosis is beneficial for the EM physician. Pulmonary embolism cannot be definitively diagnosed on FOCUS; however, in the appropriate clinical context, there are a number of sonographic findings that can be suggestive of PE.^3^,^5^,^6^ In particular, the presence of right atrial or right ventricular thrombus on FOCUS is highly predictive for the presence of associated PE, with up to 98% of patients with right heart thrombi experiencing PE as a complication.^7^,^8^

Right heart thrombi *in transit* generally appear as mobile, serpiginous or coiled hyperechoic structures seen within the right atrium or ventricle.^7^,^8^,^9^ These occur as embolic phenomena, typically from a peripheral deep vein thrombosis (DVT).^7^,^8^,^9^ The true incidence of thrombi *in transit* is unknown because the medical literature only reports an incidence between 3.6–23.3% of cases.^9^,^10^,^11^ However, the mortality associated with this finding is high, reported at 21–40%.^7^,^9^

A minority of intracardiac thrombi are the result of primary thrombosis formation within the heart, referred to as thrombi *in situ*. These are commonly associated with AF, but there are case reports of intracardiac thrombi *in situ* occurring in association with indwelling catheters or pacemaker wires, or due to low-flow states such as cardiogenic shock.^12^,^13^,^14^

Given the associated high risk of submassive or massive PE and the high mortality associated with this sonographic finding, management of right heart thrombi typically includes anticoagulation, thrombolysis, or thrombectomy.^9^

## Presenting concerns and clinical findings

The patient is a 78-year-old male with a complicated medical history including chronic hypoxemic respiratory failure from COPD on 3–4L of oxygen at baseline, HFpEF (EF 45–50%), AF, aspergillus and mycobacterium avium complex (MAC) pneumonia. He presented to the ED via EMS with palpitations and dizziness. He reported awakening with a rapid heart rate and became very dizzy and lightheaded upon sitting up. He tried taking an extra dose of sotalol as previously directed by his established cardiologist, but afterwards was still too dizzy to stand, which prompted him to call 911. He also reported that he underwent a Watchman procedure approximately six months prior to this visit and was taken off his anticoagulation for AF about four and a half months prior. Upon arrival to the ED, he was afebrile, heart rate was 112 with AF, and oxygen saturation was 95% on 5 L by nasal cannula. Blood pressure was 94/61 at the time of initial evaluation. The patient appeared chronically ill and acutely dyspneic, speaking in five-six word sentences. Lung sounds were globally diminished without rales or wheezing. There was mild bilateral symmetric, nonpitting lower extremity edema. When asked to sit upright for examination, he reported becoming dizzy. Differential considerations were broad including COPD exacerbation, exacerbation of HFpEF, pleural effusion, pneumonia/pneumonitis, PE, acute coronary syndrome, pericardial effusion, and symptomatic anemia. Laboratory diagnostics including complete blood count, comprehensive metabolic panel, and magnesium levels were ordered and found unremarkable. Troponin was within normal limits, and BNP was mildly elevated but unchanged from baseline. Electrocardiogram demonstrated AF with a ventricular rate of 107 with no other acute findings. Chest x-ray demonstrated pulmonary hyperexpansion without infiltrate or pneumothorax.

## Significant findings

### Point-of-care cardiac ultrasound

**Image 1:**
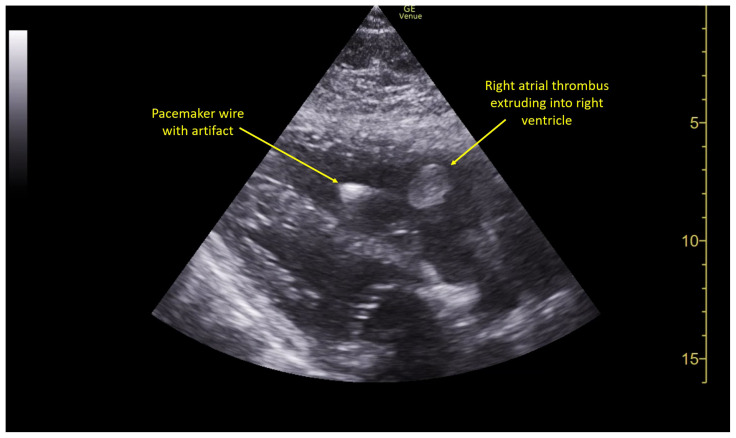
Right atrial thrombus seen extruding through tricuspid valve into the right ventricle

**Image 2:**
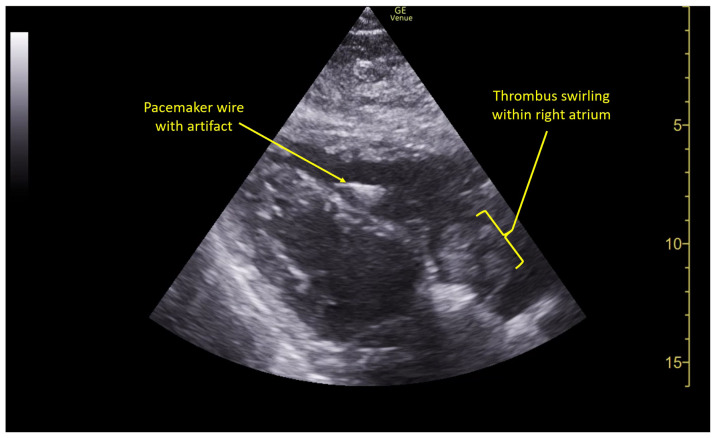
R atrial thrombus seen swirling within the right atrium US Video Link: https://youtu.be/CVSdp6oZxbQ

Pulmonary POCUS was performed by the ED physician (GE Venue, C1-5-RS 5MHz curvilinear transducer), and lung examination was unremarkable with no pleural effusion, pneumothorax, or infiltrate. Subxiphoid views (GE Venue, 3Sc-RS 4MHz phased-array transducer) were obtained because this patient’s COPD with severe pulmonary hyperexpansion made parasternal and apical 4-chamber views suboptimal. A large thrombus can be seen within the right atrium (movie 1, images 1, 2). This has a serpiginous, rounded appearance and is mobile, appearing to swirl within the right atrium with intermittent extrusion through the tricuspid valve. A pacemaker wire is also visible within the right ventricle as a non-moving, hyperechoic, linear structure with posterior enhancement artifact. Pericardial effusion is not present.

### CT angiography of the chest

**Image 3:**
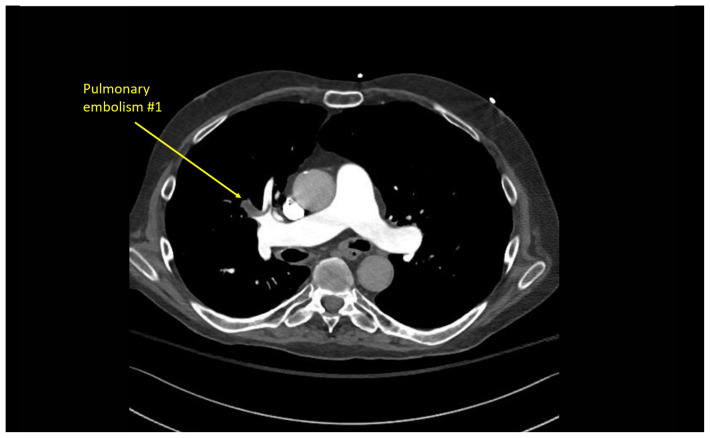
Segmental pulmonary embolism #1

**Image 4:**
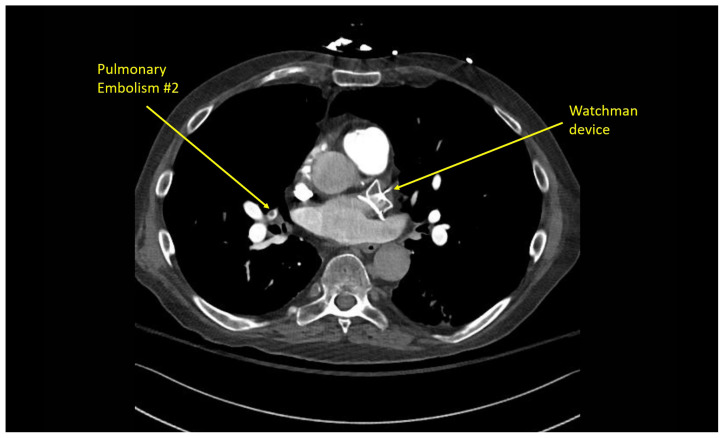
Segmental pulmonary embolism #2

**Image 5:**
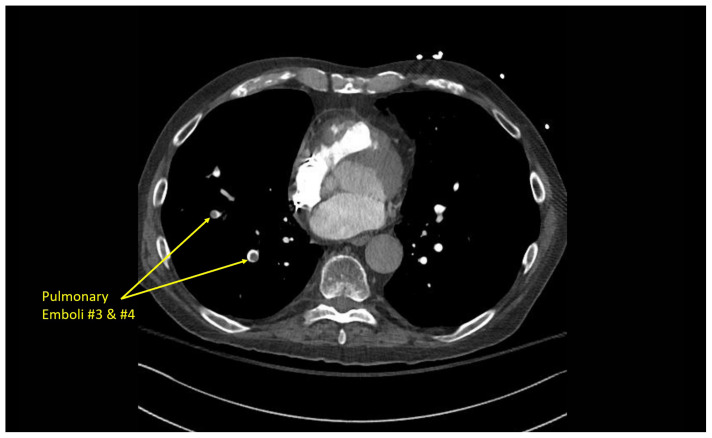
Segmental pulmonary emboli #3 and #4

**Image 6:**
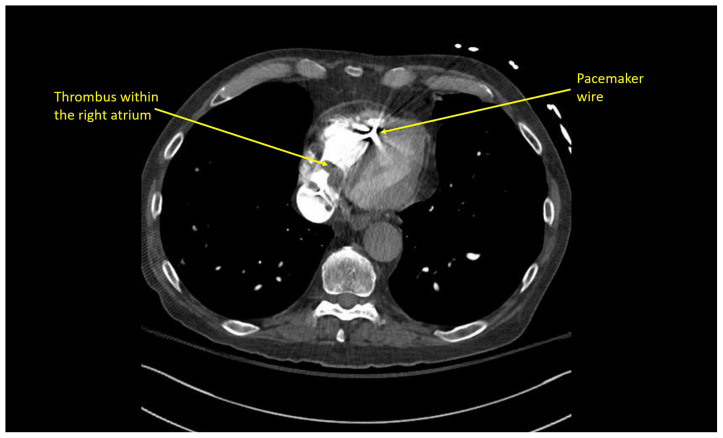
Thrombus visible within right atrium

**Image 7:**
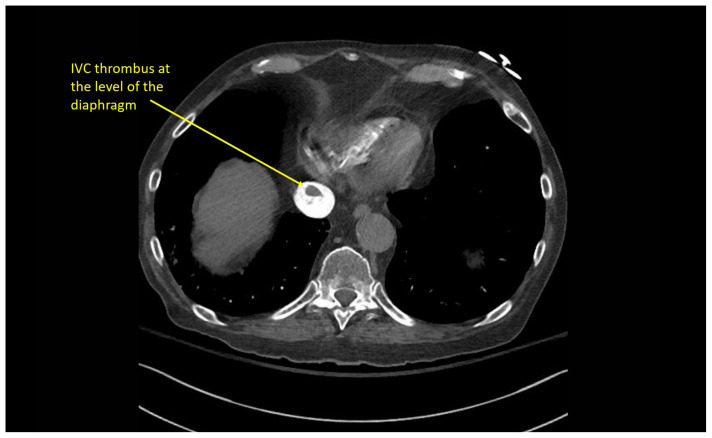
Thrombus extending into IVC at the level of the diaphragm

**Image 8:**
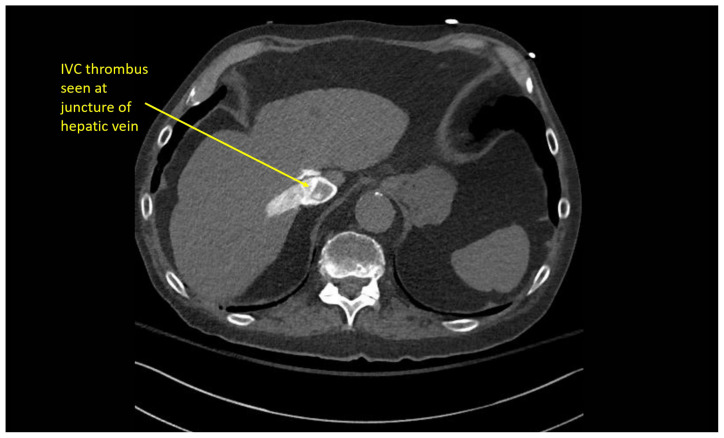
Thrombus extending caudally within the IVC

**Image 9:**
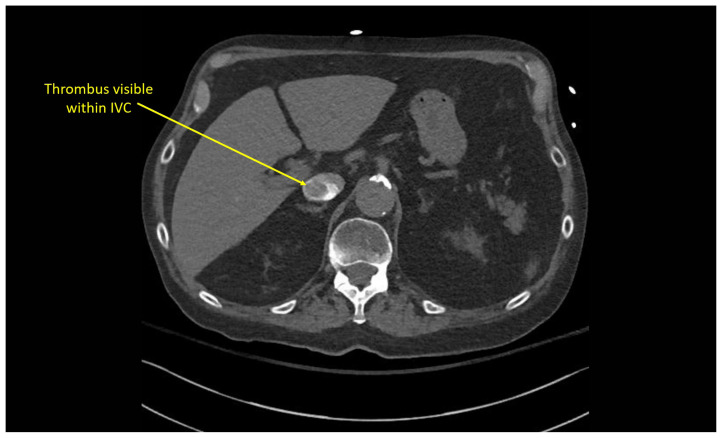
Thrombus extending caudally within the IVC CT Video Link: https://youtu.be/AsTNVTL3sJg

Multiple hypodense filling defects are present within the opacified pulmonary arteries consistent with segmental PE (images 3–5), as a complication of the thrombus extending from the right atrium (image 6) caudally into the inferior vena cava (images 7–9). The pacemaker wire is seen as a linear, hyperdense structure within the right ventricle (images 1, 2, 6). The Watchman device (image 4) can also be seen as a hyperdense, curvilinear structure within the right atrial appendage. The entire CT scan is included for review (movie 2).

## Patient course

The patient was started on heparin infusion and admitted with cardiac and hematology consultation. Thrombectomy and thrombolysis were considered, but given the patient’s multiple comorbidities, conservative management with anticoagulation alone was continued. Repeat imaging was obtained on hospital day six, at which time the IVC thrombus was no longer present, and an IVC filter was placed by interventional radiology. No complications from anticoagulation therapy arose during admission. The patient was switched to subcutaneous enoxaparin. Unfortunately, this patient was judged to have poor rehabilitation potential due to his multiple comorbidities and the fact that he was unable to ambulate with a walker. He was discharged to a skilled nursing facility on hospital day eight. He re-presented to the ED approximately five weeks later with unrelated complaints, was doing well on anticoagulation, and follow-up CT demonstrated interval resolution of his pulmonary emboli and intracardiac thrombus.

## Discussion

Right intracardiac thrombi are a relatively uncommon sonographic finding on FOCUS. However, the presence of this finding is highly associated with concomitant submassive/massive PE and portends a worse overall prognosis, with mortality rates reported between 21–40%.^7^,^9^ The true incidence of right heart thrombi is not known. In patients with diagnosed PE, the incidence of intracardiac thrombi are reported in only between 3.6 and 23.3% of cases; however, selection bias in studies including only high-risk PE likely skews this estimate upwards of its true incidence.^9^,^10^,^11^

In the case presented, the etiology of thrombosis is likely multifactorial. The patient had multiple risk factors for thromboembolism, including older age, frequent hospitalizations, minimal activity, and the presence of indwelling intravascular devices. This patient had a large thrombus extending from the right atrium into the inferior vena cava. It is unclear whether the patient’s pacemaker wire may have been a nidus for thrombosis, or whether his underlying AF and HFpEF resulted in enough atrial stasis to allow thrombus formation. It is plausible that his recent cessation of anticoagulation following his Watchman procedure may have contributed as well.

Patients with right heart thrombi can present with a myriad of symptoms, most of which mirror those of pulmonary emboli. The most common presenting symptoms include chest pain, dyspnea, and syncope.^15^ The diagnosis of intracardiac thrombosis can be confirmed with either CT or echocardiography. Computed tomography has been demonstrated to have higher sensitivity for the detection of intracardiac thrombi than transthoracic echocardiography, but is equivalent or inferior to transesophageal echocardiography (TEE).^16^ In an ED setting, TEE may be challenging to obtain because the sedation required to perform this procedure may be contraindicated in hemodynamically unstable patients. Computed tomography does have some disadvantages; it requires the use of ionizing radiation and contrast materials, which can limit the use of CT in patients with contrast allergy or renal dysfunction. Moving patients with hemodynamic compromise to the radiology suite is often precarious because there are limited resources to provide ongoing resuscitation. Focused cardiac ultrasonography offers ED clinicians a reasonable alternative because formal echocardiography and CT imaging may not be available at all times, and may not be available at all in smaller hospitals or freestanding emergency departments.

The sonographic appearance of intracardiac thrombi is variable, depending on the size and origin of the clot. Thrombi *in transit* (Type A thrombi) are typically embolic in nature and tend to be longer, thinner, and appear serpiginous or coiled.^17^ These are mobile and may have a swirling appearance, and may move in and out of the plane of view. Thrombi *in situ* (Type B thrombi) develop within the cardiac structures as a result of a low-flow state.^17^ These tend to be more rounded or globular in shape and are less mobile in appearance. These are morphologically similar to thrombi that develop in the left heart in association with AF.^17^ Type C thrombi have a combination of both features.^17^ This patient likely had a type C thrombus because he had elements of both types. The presence of his pacemaker lead and AF may have created conditions favoring thrombosis, which then propagated down into the inferior vena cava and allowed the extrusion of mobile thrombus into the right atrium. The patient also had negative bilateral lower extremity doppler ultrasound studies, reducing the likelihood of a purely embolic phenomenon.

Focused cardiac ultrasonography does offer the clinician some ability to diagnose hemodynamically significant PE as well. As discussed in the introduction, FOCUS cannot be used to rule out the diagnosis of intracardiac thrombi or PE, especially in low-risk patients. 3 The right ventricle (RV) is relatively thin-walled and compliant relative to the left ventricle (LV), and the pressures in the right heart and pulmonary vasculature are substantially lower than that of the LV and systemic circulation.^18^,^19^ Focused cardiac ultrasonography does not directly image PE burden, but rather allows the clinician to assess for the hemodynamic impacts of right heart outflow obstruction on the visible structures of the heart and inferior vena cava. Given this, it is understandable that literature suggests that FOCUS is most useful in patients with larger and more centrally located thromboemboli.^20^ Findings associated with PE include indicators of right heart strain, such as an increased RV:LV ratio, McConnell’s sign, a D-shaped left ventricle or “D-sign,” and a tricuspid annular plane systolic excursion (TAPSE) <1.7 cm.^3^,^21^

Dilation of the right ventricle with bowing of the ventricular septum towards the left ventricle (the “D-sign,”) obtained in the parasternal short axis view) (images 10,11) can be indicative of increased right heart pressure associated with right heart outflow obstruction.^22^ In the setting of right heart strain, the right heart volume may exceed the volume of the left heart, resulting in an increased RV:LV ratio.^22^

Tricuspid annular plane systolic excursion (TAPSE) is a measurement of the tricuspid valve displacement that occurs with the longitudinal shortening during contraction of the RV, and is best measured in the apical-4 chamber view using M-mode. 23 TAPSE has been shown to correlate with RV ejection fraction, making it useful in the evaluation of acute pulmonary embolism, and has been shown to have predictive value regarding the prognosis of these patients.^24^ However, TAPSE is not specific to PE, and is also useful in assessment of chronic conditions such as pulmonary hypertension, congestive heart failure, and cardiomyopathy.^24^ In this patient, TAPSE and D-sign could not be assessed because the parasternal and apical 4-chamber views were unobtainable due to severe pulmonary hyperexpansion from underlying COPD.

Differentiation of acute right heart strain due to PE from chronic pulmonary hypertension can be challenging because these conditions share echocardiographic findings of right ventricular dysfunction.^18^ In chronic processes such as pulmonary hypertension, the longstanding increased right pressures result in hypertrophy and thickening of the RV wall. However, in the setting of an acute right heart outflow obstruction such as PE, the pressure in the right heart increases causing the RV to enlarge, but the wall of the right heart remains thin.^18^ Measurement of the RV free wall can therefore help differentiate between acute and chronic right heart strain. An RV free wall thickness ≤ 5mm is suggestive of an acute process, whereas an RV free wall thickness of > 5mm is more consistent with a chronic process.^18^

Benefits of FOCUS for the evaluation of intracardiac thrombi and PE are many. Its immediate availability and portability allows the clinician to rapidly evaluate critically ill and unstable patients without requiring transport to a radiology department where ongoing resuscitative measures may be limited or unobtainable. It allows real-time assessment of volume status and myocardial function, allowing the clinician to quickly exclude other potential causes of the shock state. POCUS is also more readily accessible than traditional radiology exams, allowing the patient to receive more timely care. In low-resource or rural settings where access to formal radiology may be unavailable, the increasing utilization of point-of-care and handheld ultrasound devices have improved diagnostic capabilities substantially.

However, limitations of POCUS for the evaluation of intracardiac thrombus and PE are also important to consider. POCUS is both operator dependent and can be limited by the body habitus of the patient. FOCUS is less sensitive for the diagnosis of intracardiac thrombi than TEE or CT imaging. Likewise, FOCUS has poor overall sensitivity for PE, particularly for smaller, more distal emboli with minimal or no hemodynamic impact. As such, FOCUS should not be used to rule out the diagnosis of PE, particularly in stable, low risk patients, but to help risk stratify the patient with suspected submassive or massive PE.^3^,^32^

Presence of a mobile, serpiginous mass on FOCUS is highly specific for the diagnosis of intracardiac thrombi, but in rare cases, large vegetations may be mistaken for thrombi.^7^ Differentiating intracardiac thrombi from other intracardiac masses can also be challenging. Primary cardiac tumors are quite rare, with an incidence of 0.002% – 0.3%, including benign cardiac tumors isuch as myxomas, lipomas, and fibromas, and primary malignant tumors including sarcomas and leiomyomas.^25^,^26^ Metastatic spread to the heart is much more common, occurring at a rate approximately 40 times that of primary cardiac tumors.^27^ While FOCUS and standard transthoracic echo commonly identify the presence of intracardiac masses, these are often inadequate to further characterize intracardiac tumors.^28^ Contrast-enhanced transthoracic or transesophageal echocardiography can be helpful in distinguishing thrombi from tumors because tumors are typically highly vascularized whereas thrombi are avascular.^27^,^28^ Magnetic resonance imaging is widely considered to be superior to ultrasonography for the definitive diagnosis of cardiac tumors.^26^

Sonographic findings such as right heart dilatation are not specific to PE, and may also be present in patients with other disease states that produce increased pulmonary artery pressures, such as pulmonary hypertension, obstructive sleep apnea, or right heart failure.^3^ When feasible, confirmation of the diagnosis of intracardiac thrombosis and PE should be performed with CT angiography. In cases where the patient is peri-arrest or too unstable to transport for formal imaging, FOCUS can be used to guide therapy.

Management of intracardiac thrombi typically requires initiation of anticoagulation, but given the high mortality associated with this condition, thrombolysis or mechanical thrombectomy are often considered.^8^,^15^ The data on this topic are limited to case series and case reports, so there is substantial bias regarding which patients underwent which intervention, resulting in a lack of high-quality data to guide therapy. Similarly, much of the literature precedes the development of modern percutaneous interventional techniques. More recent literature suggests that percutaneous thrombectomy is emerging as a reasonable alternative to surgical thrombectomy.^29^,^30^,^31^

Focused cardiac ultrasonography is a powerful bedside tool for the diagnosis of intracardiac thrombi in ED patients. The presence of intracardiac thrombus is highly suggestive of associated PE, and patients with this finding have high rates of mortality. This sonographic finding should prompt further evaluation for PE, and consideration of anticoagulation versus thrombolysis or thrombectomy.

## Supplementary Information
