# Supplementary figures and images for: A Case Report of Right Atrial Thrombosis Complicated by Multiple Pulmonary Emboli: POCUS For the Win!
Source: J Educ Teach Emerg Med. 2025 Jan 31;10(1):V1–V11. doi: 10.21980/J8TM07 (PMC11801490; doi:10.21980/J8TM07)

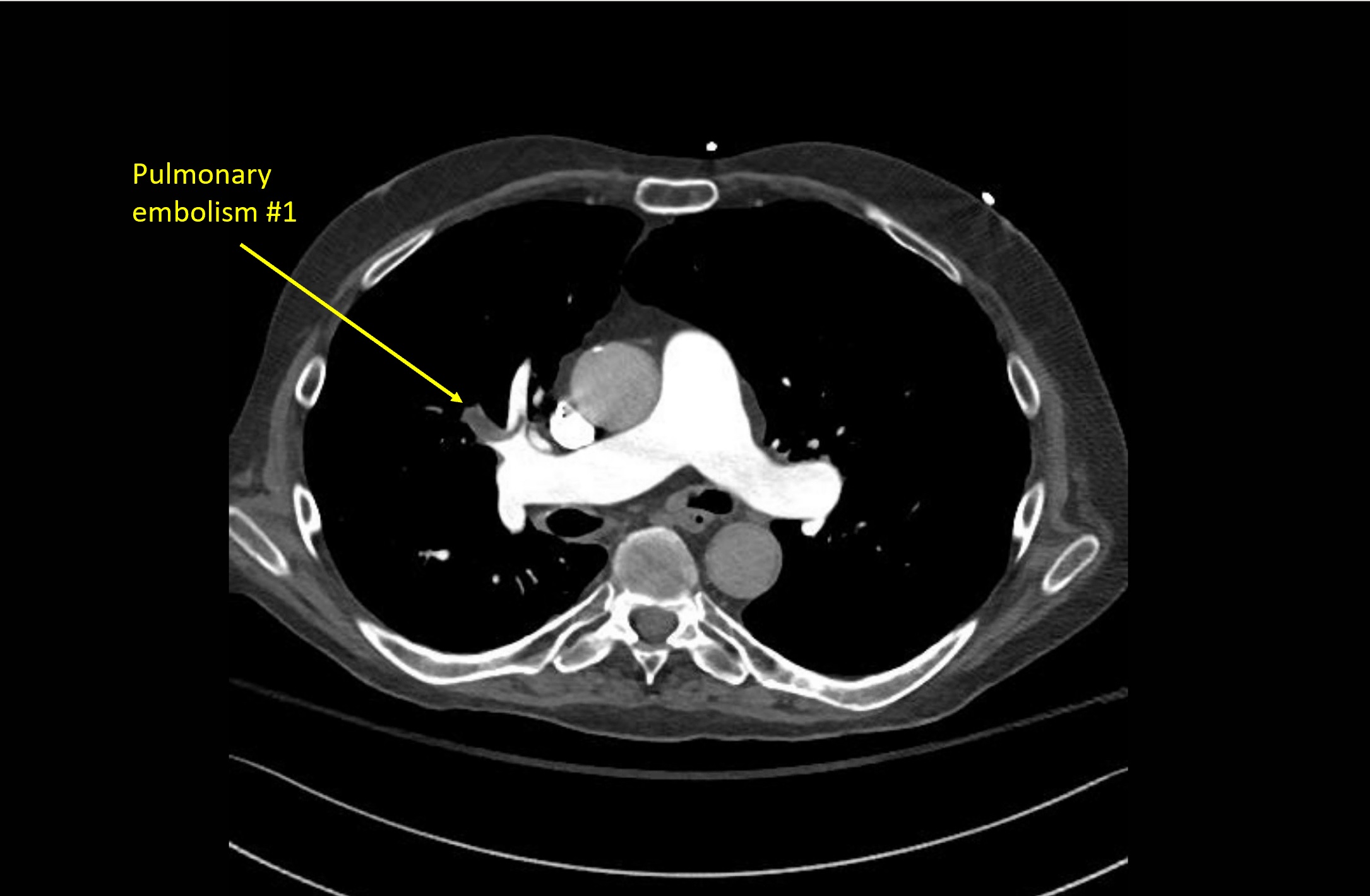

Supplement: Supplementary file 1 [file 10-1-V1-supp1.JPG]

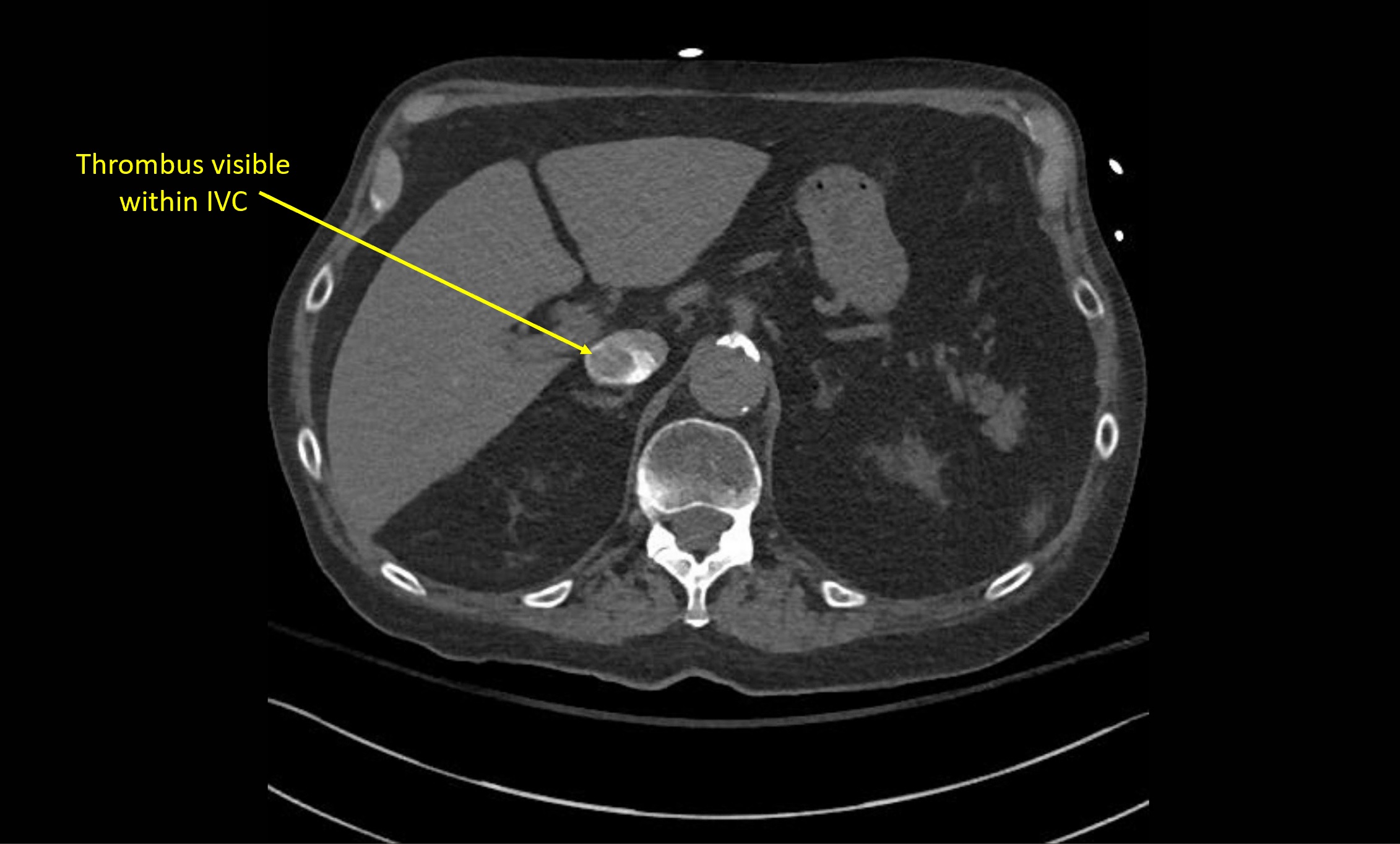

Supplement: Supplementary file 2 [file 10-1-V1-supp2.jpg]

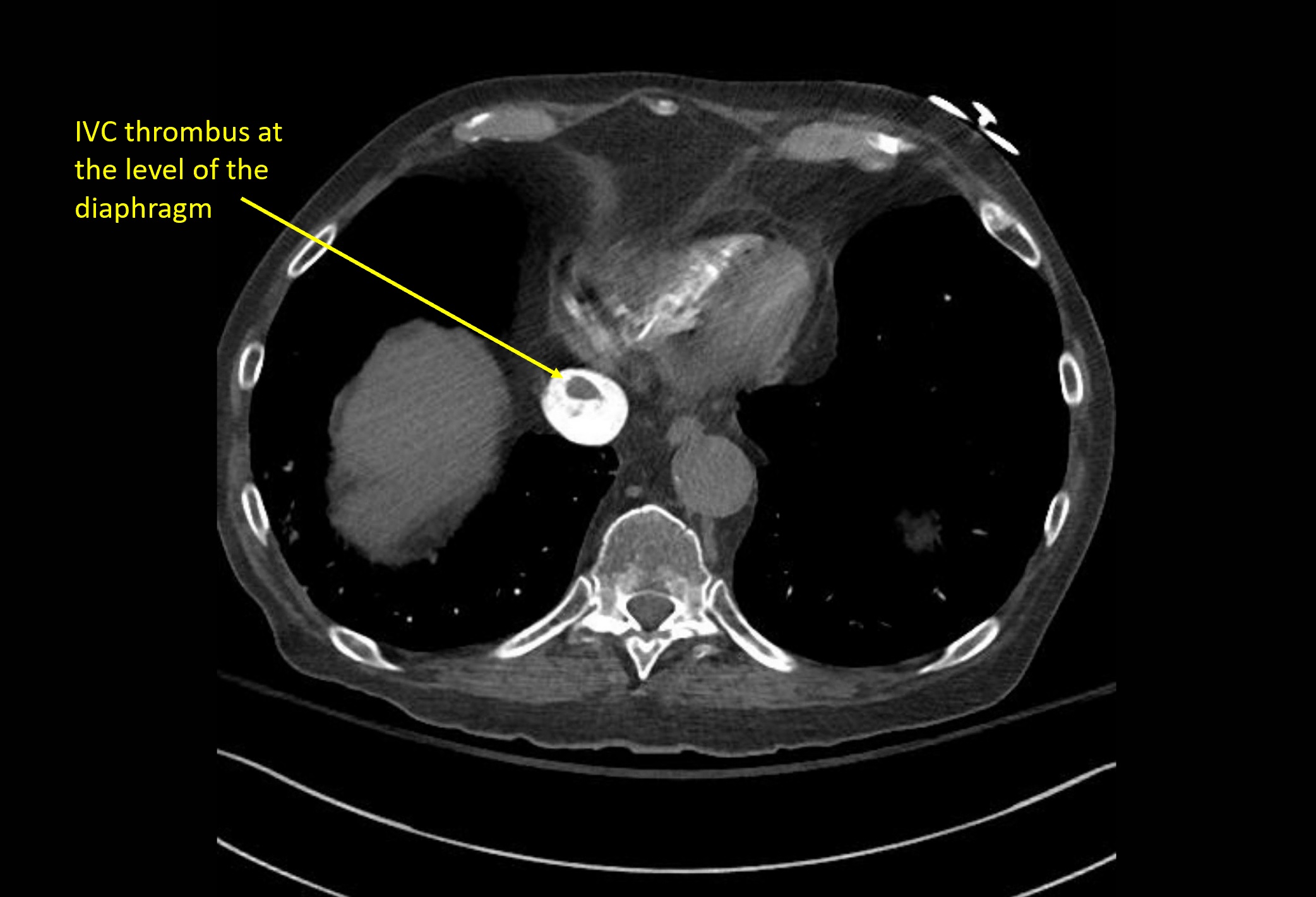

Supplement: Supplementary file 3 [file 10-1-V1-supp3.jpg]

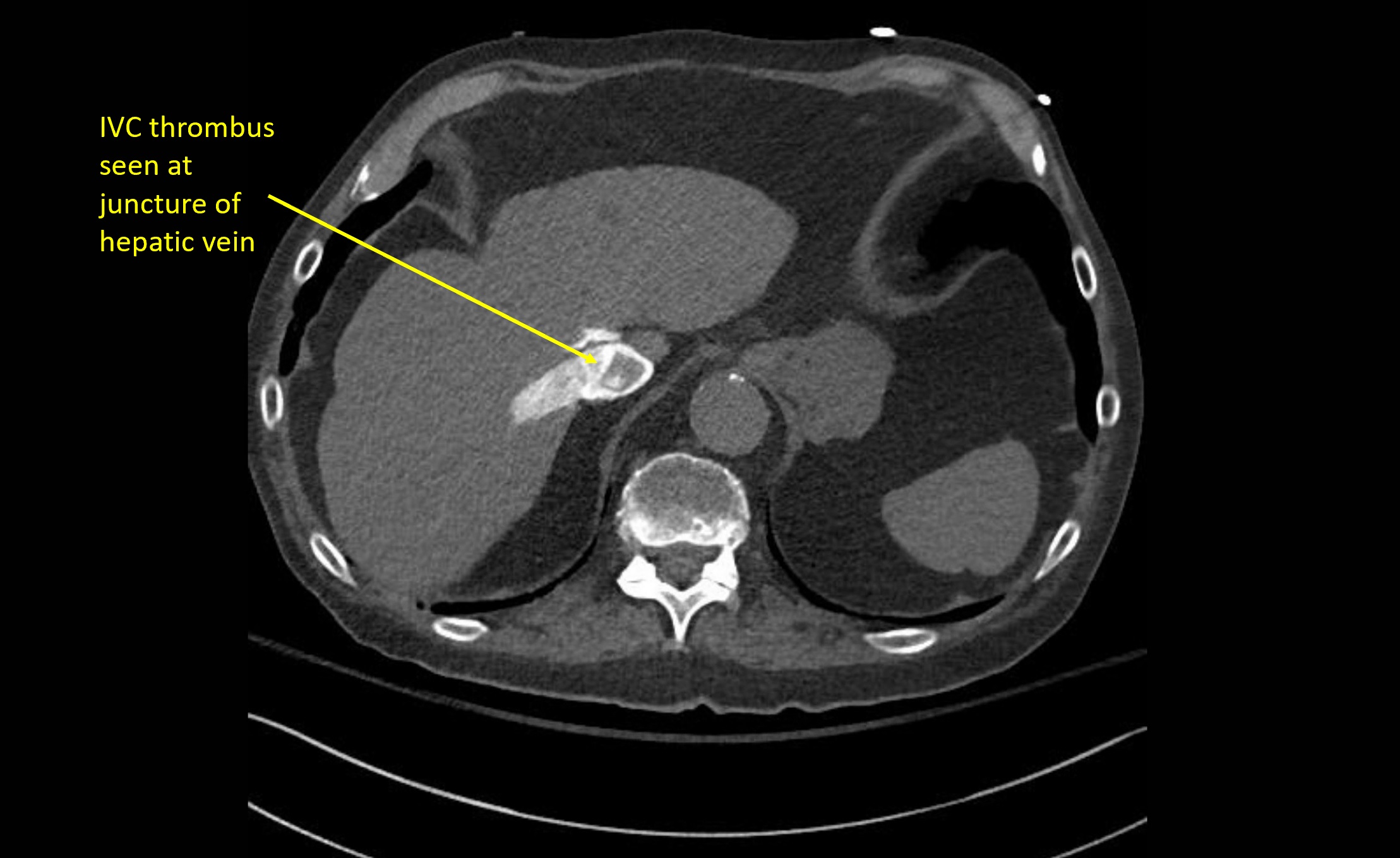

Supplement: Supplementary file 4 [file 10-1-V1-supp4.jpg]

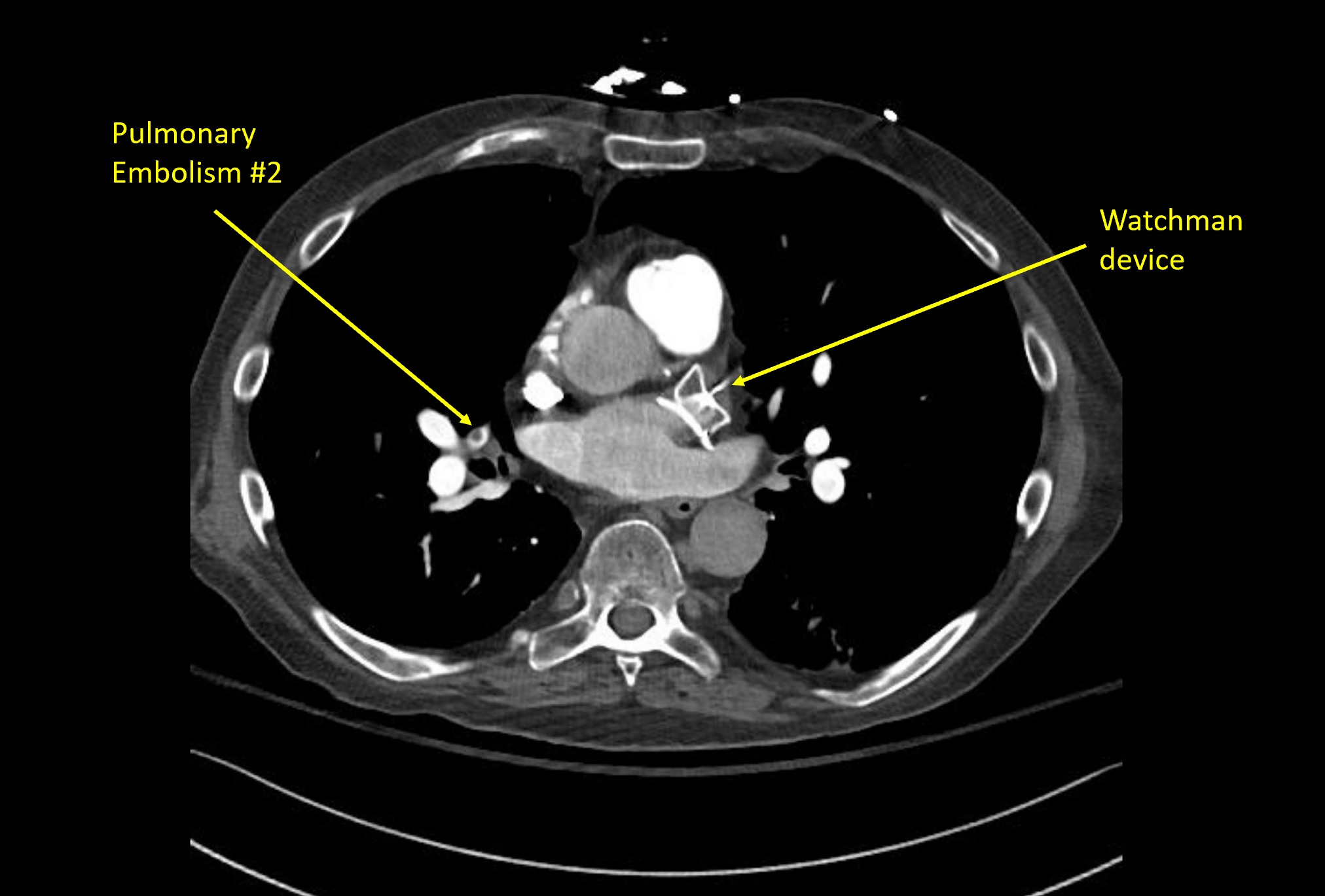

Supplement: Supplementary file 5 [file 10-1-V1-supp5.JPG]

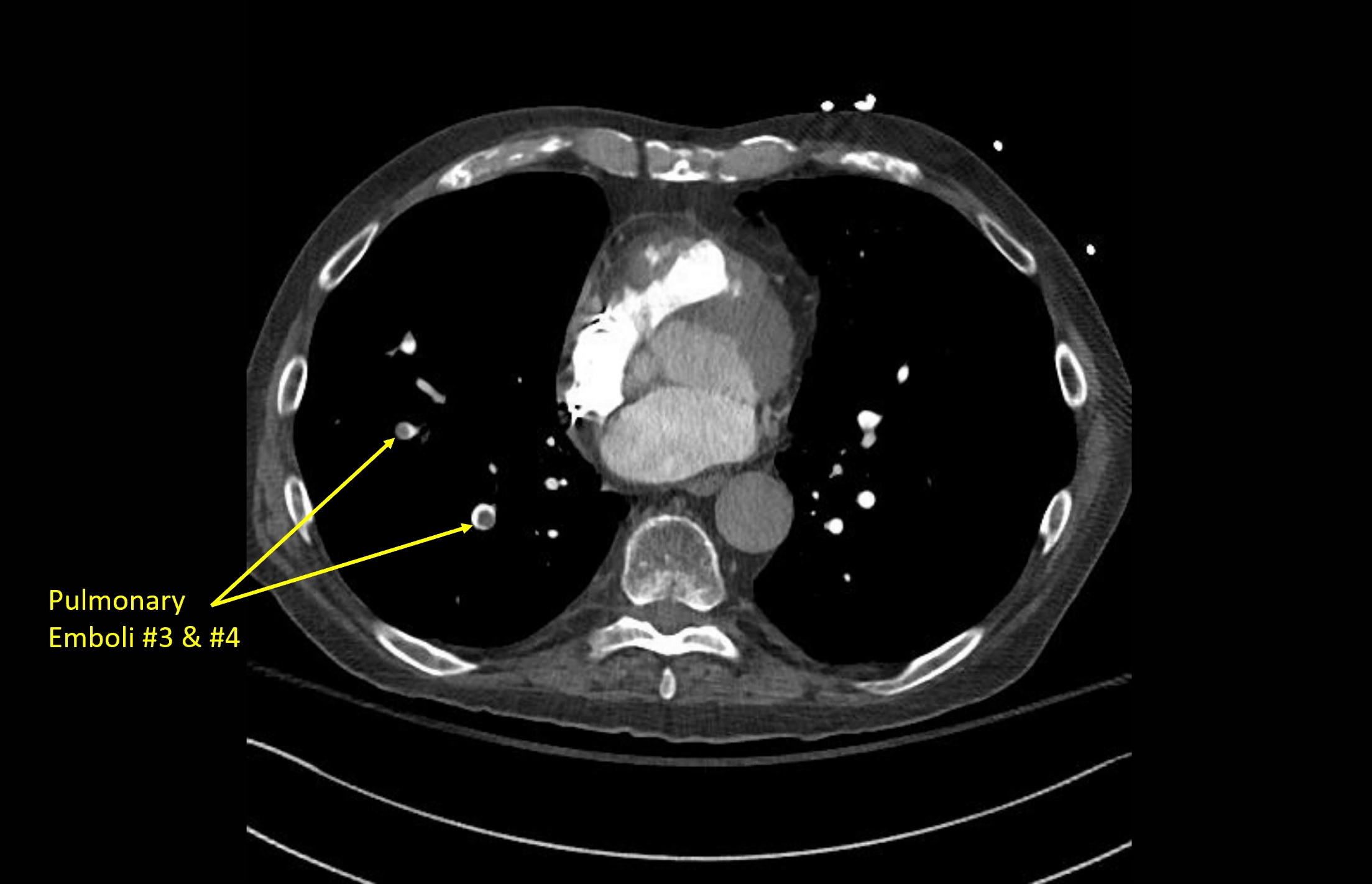

Supplement: Supplementary file 6 [file 10-1-V1-supp6.JPG]

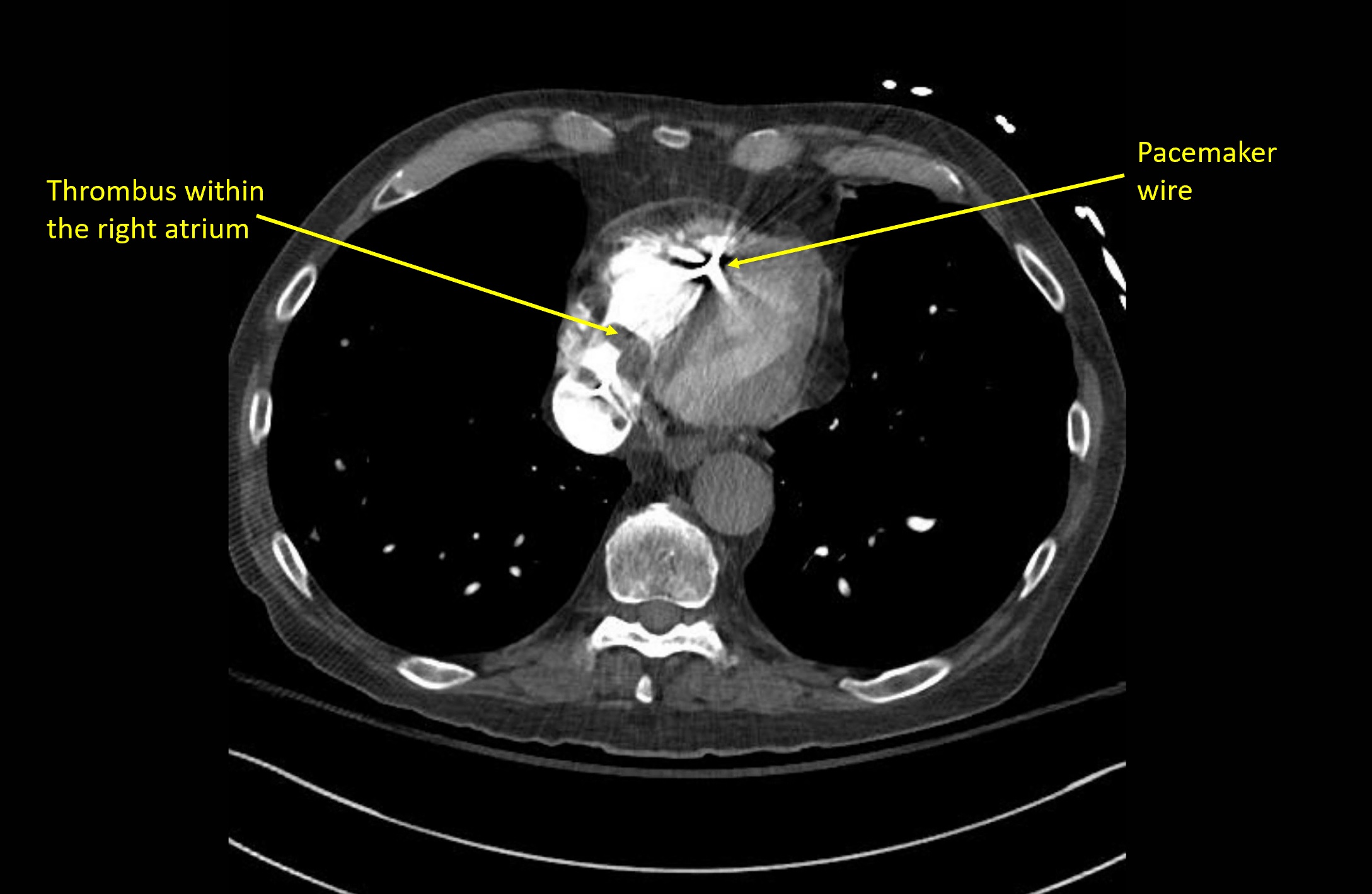

Supplement: Supplementary file 7 [file 10-1-V1-supp7.jpg]

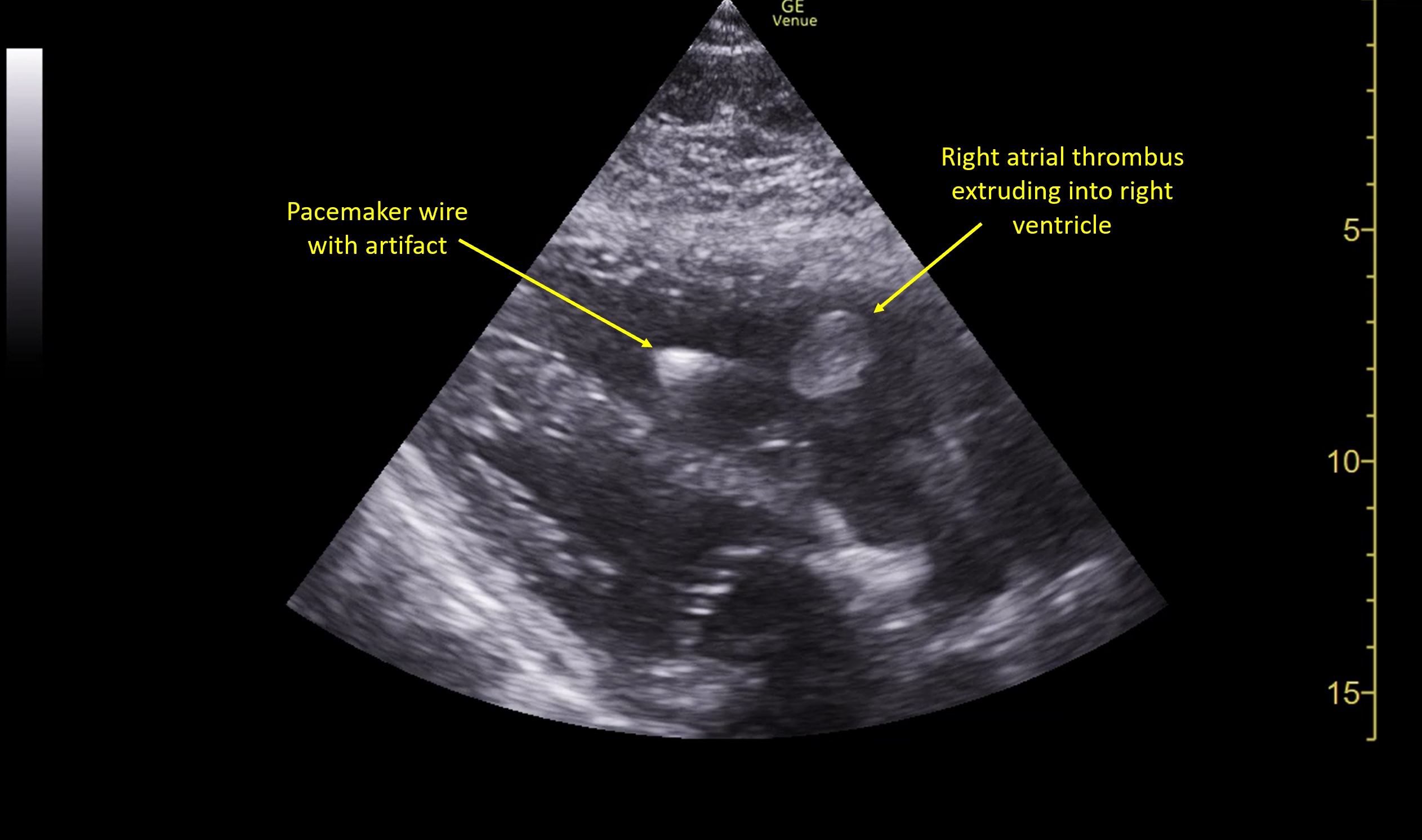

Supplement: Supplementary file 9 [file 10-1-V1-supp9.jpg]

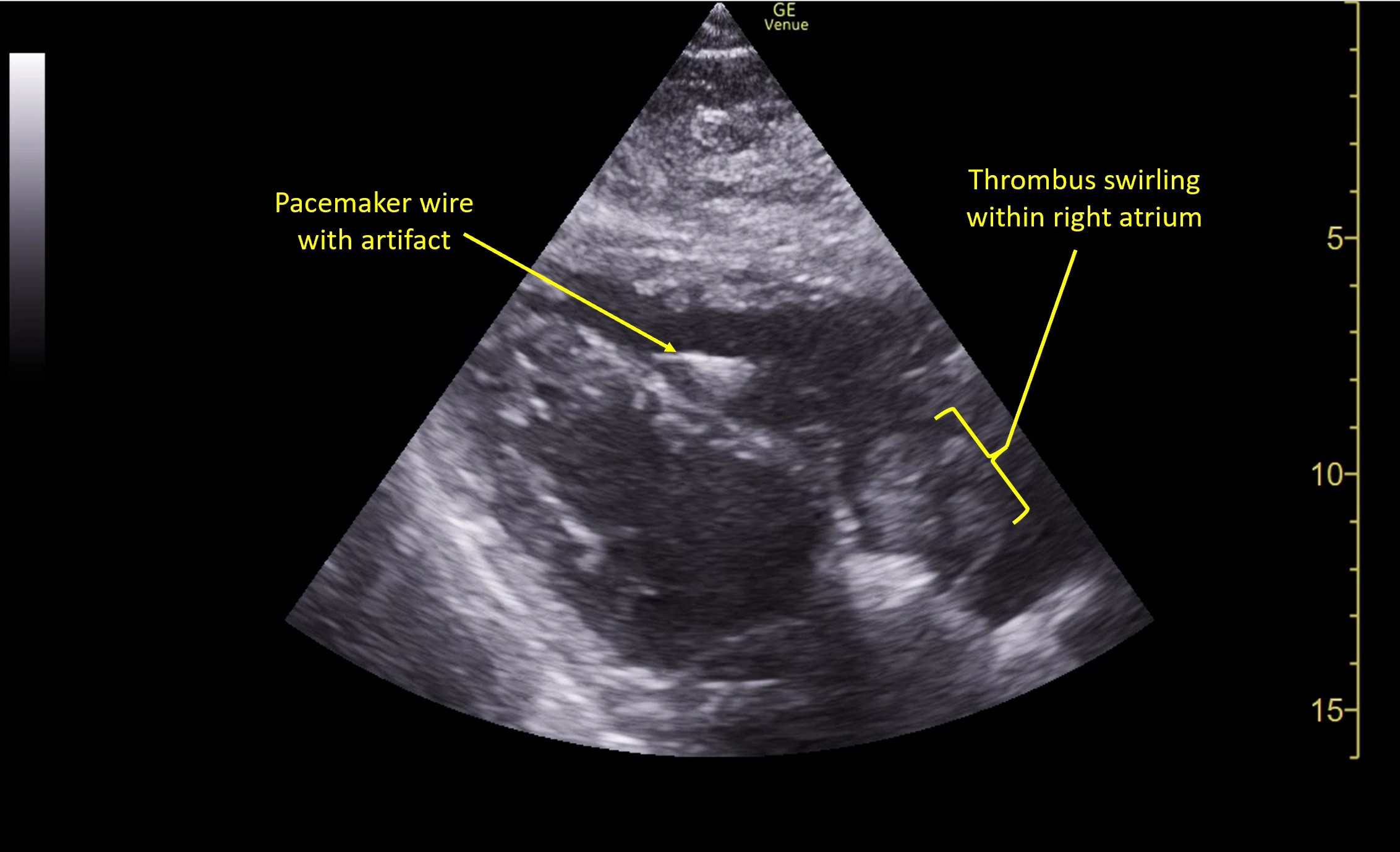

Supplement: Supplementary file 11 [file 10-1-V1-supp11.jpg]

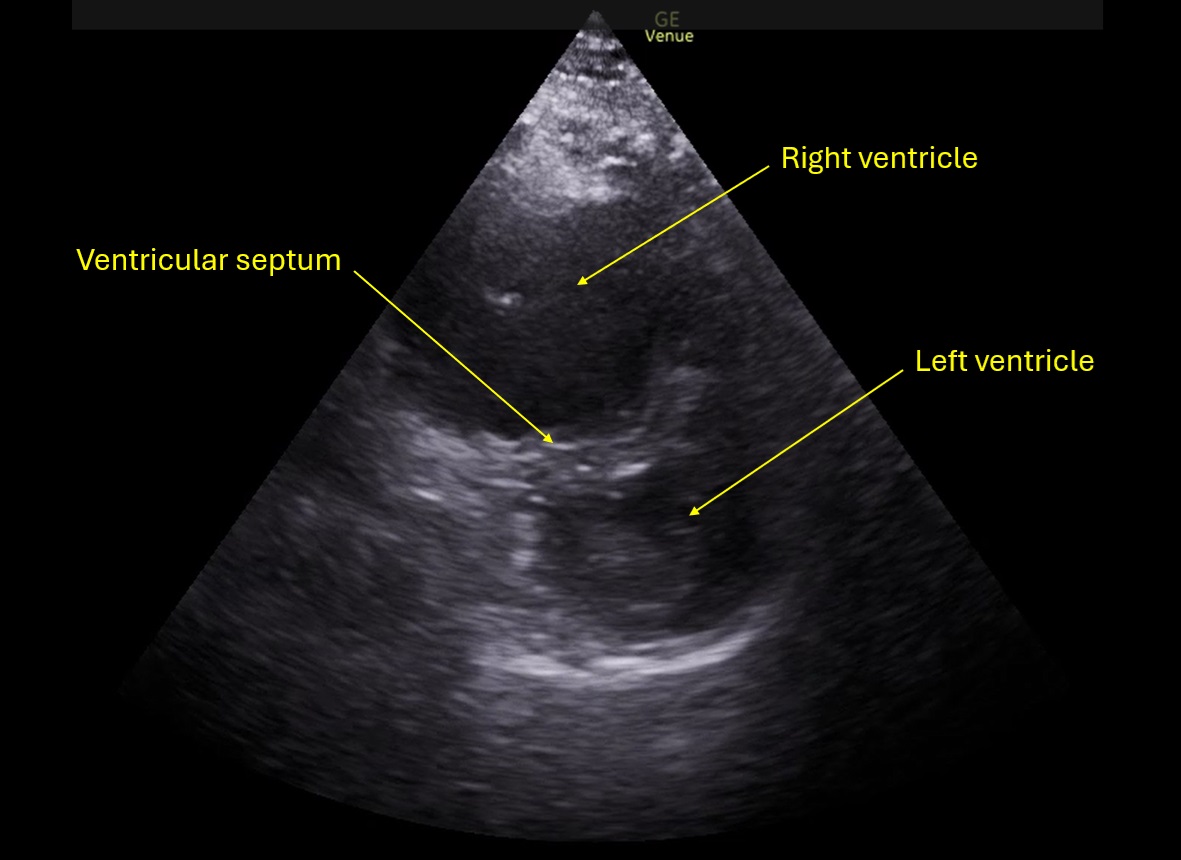

Supplement: Supplementary file 13 [file 10-1-V1-supp13.jpg]

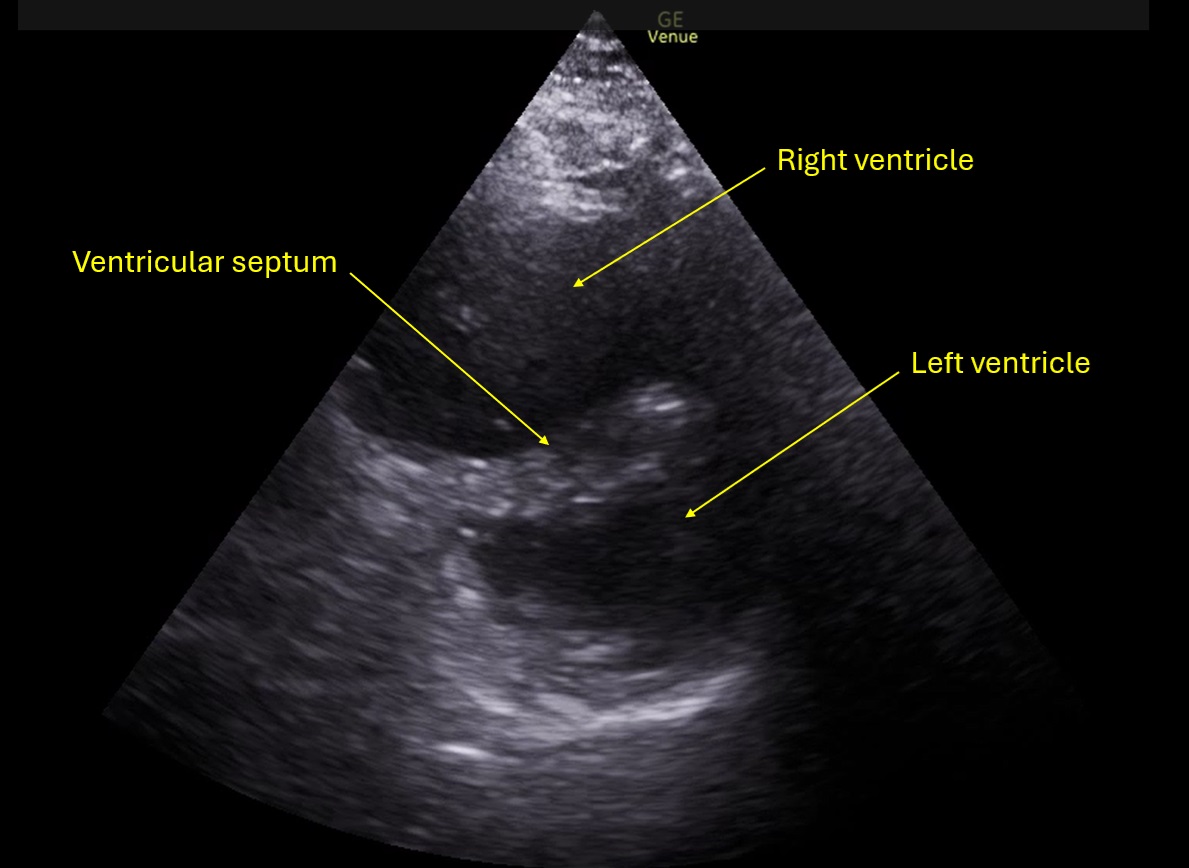

Supplement: Supplementary file 14 [file 10-1-V1-supp14.jpg]
